# Investigation of Inhibition Mechanism of Chemokine Receptor CCR5 by Micro-second Molecular Dynamics Simulations

**DOI:** 10.1038/srep13180

**Published:** 2015-08-24

**Authors:** Ramin Ekhteiari Salmas, Mine Yurtsever, Serdar Durdagi

**Affiliations:** 1 Department of Chemistry, Istanbul Technical University, Istanbul, Turkey; 2 Department of Biophysics, School of Medicine, Bahcesehir University, Istanbul, Turkey

## Abstract

Chemokine receptor 5 (CCR5) belongs to G protein coupled receptors (GPCRs) and plays an important role in treatment of human immunodeficiency virus (HIV) infection since HIV uses CCR5 protein as a co-receptor. Recently, the crystal structure of CCR5-bound complex with an approved anti-retroviral drug (maroviroc) was resolved. During the crystallization procedure, amino acid residues (i.e., Cys224, Arg225, Asn226 and Glu227) at the third intra-cellular loop were replaced by the rubredoxin for stability reasons. In the current study, we aimed to understand the impact of the incorporated rubredoxin on the conformations of TM domains of the target protein. For this reason, rubredoxin was deleted from the crystal structure and the missing amino acids were engineered. The resultant structure was subjected to long (μs) molecular dynamics (MD) simulations to shed light into the inhibitory mechanism. The derived model structure displayed a significant deviation in the cytoplasmic domain of TM5 and IC3 in the absence of rubredoxin. The principal component analyses (PCA) and MD trajectory analyses revealed important structural and dynamical differences at apo and holo forms of the CCR5.

Chemokine proteins belong to the G protein-coupled receptor (GPCR) family^1^,^2^. GPCRs have seven trans-membrane (TM) domains which are snaked through membrane bilayer. Their cytoplasmic sides are linked to intracellular guanosine nucleotide-binding protein (G-protein) to transfer cellular signaling^3^. An antagonist that targets the CCR5 in the inactive state may inhibit the transmission of intracellular signaling^4^. However in the active state, they are stabilized to become eligible for intracellular signal transduction^4^. The X-ray structure of a few GPCR targets have been solved recently in both antagonist-bound inactive states^5^,^6^,^7^,^8^ and agonist-bound active states^9^,^10^,^11^ The chemokine proteins were classified into four classes (C, CC, CXC and CX3C) with respect to the number and position of Cysteine residues in the amino acid sequence^12^,^13^,^14^,^15^. The CC chemokine receptor type 5 (CCR5) plays a significant role in the HIV infection process^4^. HIV proteins mainly use CCR5 or CXCR4 as co-receptors to enter into the cell. The inhibitor of CCR5 (maraviroc; brand-named *selzentry*, or *celsentri* outside the U.S.A.) was approved by FDA in 2007^4^. Maraviroc is a non-peptide HIV fusion inhibitor. A number of small ligand CCR5 inhibitors were determined to have strong antiviral influences both in *in vitro* and clinical experiments^16^. For example, TAK-779 was suggested before the maraviroc as CCR5 inhibitor^17^,^18^. Recently, the crystal structure of CCR5 in the presence of the anti-HIV drug maroviroc was successfully crystallized with resolution of 2.7 Å^19^. This achievement stimulated the anti-HIV drug development studies.

The V3 loop of envelope glycoprotein (gp120) is critical in HIV fusion when it links to the co-receptor^20^. Extra cellular (EC) domains of CCR5 participate at the gp120-CCR5 coupling^20^,^21^,^22^. Although chemokine receptors can function as viral co-receptors, the CCR5 target is one of the most physiologically important co-receptor during natural infection^23^. Despite of many potential investigations on HIV field, many questions regarding the interactions between gp120 and CCR5 is still not solved: How gp120 uses CCR5 as a co-receptor to enter the human cell? What is the molecular mechanism of this event? How anti HIV drugs in the position of CCR5 inhibitors protect the coupling of gp120 and EC domains of the CCR5? So far, most of the *in silico* studies on CCR5 were based on homology models^24^,^25^,^26^,^27^. Recently, Tamamis *et al.*^28^ used the CCR5 X-ray structure for investigation of the molecular recognition of CCR5 by a dual tropic HIV-1 gp120 V3 loop via short (20 ns) MD simulations. In the present study, we aimed to understand better the molecular mechanism of CCR5-drug interactions via long (0.6 μs in total) MD simulations. The analyses such as principle component analysis (PCA) and clustering approach were performed to eliminate the insignificant dynamic modes and the conformational changes throughout the simulations and to explore the structural differences between the apo and holo forms of the CCR5.

## Methods

### The Modeling of apo and holo Proteins

**Figure 1:**
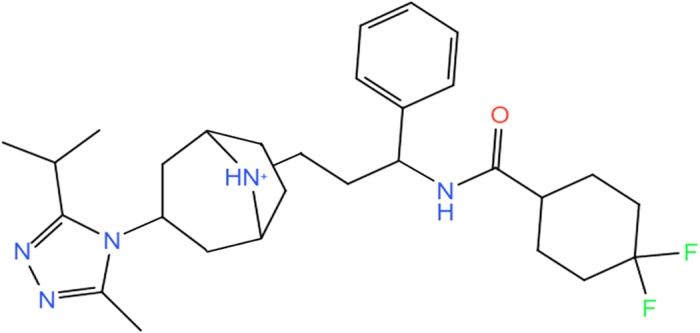
Chemical structure of maraviroc used as co-crystallized ligand in holo system.

**Figure 2:**
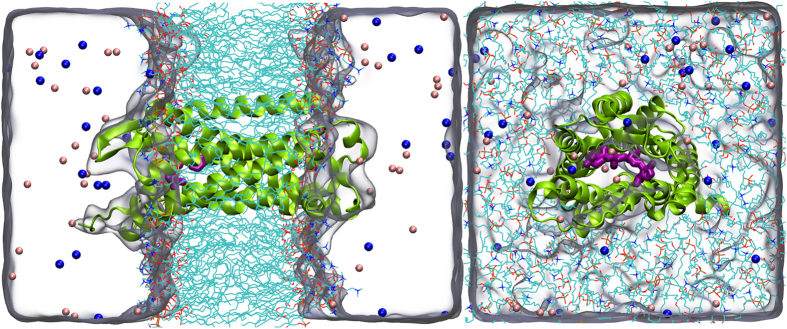
Simulated systems which is consist of embedded CCR5 chemokine (CCR5 is represented as a green cartoon) complex into lipid bilayer (DPPC, shown with stick representation), waters (shown as quick surface) and counter ions (K^+^/Cl^−^, shown with blue and pink balls, respectively).

The crystal structure of CCR5 chemokine receptor with a co-crystallized antagonist maraviroc (Fig. 1) with a resolution of 2.7 Å was retrieved from the protein data bank (PDB) server (PDB ID: 4MBS)^19^. The rubredoxin molecule was removed from structure and the missing amino acids Cys224, Arg225, Asn226 and Glu227 in the position of third intracellular loop (IC3) were modeled using Modeller 9.13^29^. The new CCR5 receptor structure was prepared using Protein Preparation Wizard^30^ implemented in the Schrodinger Maestro molecular modeling package. Protonation states of the target were adjusted using PROPKA^31^ at the physiological pH of 7.4. In order to compare the inhibition efficiencies of the CCR5 antagonist at the apo (ligand-free) and holo (ligand-occupied) states, both forms of the receptor were studied. Apo and holo complexes were embedded into the 1,2-dipalmitoyl-sn-glycero-3-phosphocholine (DPPC) membrane bilayer and orientation of the target protein in the lipid bilayer was adjusted using Orientations of Proteins in Membranes (OPM) server^32^. The membrane bilayers have been modeled by utilizing VMD^33^ Membrane Builder plugin tool. The CCR5 crystal structure involves 4 Cysteine residues. They form two disulfide bonds, between Cys20-Cys269 and Cys101-Cys178 residues. These essential disulfide bonds were constrained using the patch DISU of the VMD. Then, the membrane embedded proteins were solvated with TIP3P^34^ explicit water models employing the Helmut Grubmuller’s Solvate 1.0 program. Finally the systems were neutralized (by 0.15 M of KCl solution) using VMD’s Solvate plugin and located in a box having water thickness of 15 Å. The total number of atoms involved was around 58000. (Fig. 2).

### Molecular Dynamics (MD) Simulations

MD simulations were carried out using the NAMD 2.9^35^. The CHARMM36^36^ force field was used for the CCR5 membrane protein and DPPC lipid bilayer. ParamChem^37^ server was used to generate force field for maraviroc. All covalent bonds between hydrogen and heavy atoms were constrained using the SHAKE algorithm. In order to simulate the isothermal–isobaric ensemble (NPT) and control the used temperature (310 K) and the pressure (1 bar), individually, Langevin dynamics and Langevin piston methods were used. The Langevin damping coefficient and the piston period were set to 1.0 ps^−1^ and 0.05 ps, respectively. For long-range electrostatic interactions, a cut-off distance of 12.0 Å was used with Particle Mesh Ewald (PME) approach in a periodic box. A time-step of 1.0 and 2.0 fs was used for equilibration and production MD runs, respectively.

### Minimization and Constrained Equilibration

The systems were minimized and equilibrated before performing the MD production runs. This procedure was carried out as follows: i) in order to achieve the equilibrium of lipid tails, 10^4^ steps of minimization and 2.0 ns isochoric-isothermal (NVT) MD simulations with all atoms fixed except atoms of lipid tails was performed, ii) 2 × 10^4^ steps of minimization and 2.0 ns simulations under NVT conditions were used with constrained protein, iii) Final equilibration was carried out with all atoms released through 4.0 ns under NVT ensemble, (iv) Finally 0.3 μs production MD simulations under isothermal-isobaric (NPT) conditions were performed for each *holo* and *apo* states (total 0.6 μs). During the MD simulations, the coordinates of all atoms were recorded for every 10.0 ps for further analyses.

### Calculations of non-bonding Interaction Energies

The average interaction energies between the amino acid residues in the binding cavity and inhibitor were calculated using 3 × 10^4^ trajectories. NAMD energy plugin implemented in VMD and CHARMM36 force field were used for this aim. The cut-off distance and switch distance were set to 12.0 Å and 10.0 Å, respectively.

### Principal Component Analysis (PCA)

PCA^38^,^39^ is a popular statistical data-processing analysis for the decreasing of large-dimensional observations sets onto collective data. In other words, PCA is a process of determining models in data, and describing the data as to focus on their likenesses and distinctions.

### Applying PCA on MD Simulations

A standard MD trajectory involves the coordinates of overall atoms stored during the simulations. For a system of N atoms, the input data set for PCA can be a statistical data matrix and each column of the matrix indicates Cartesian coordinate for a particular atom at every frame. Before starting the PCA, each frame should be aligned respect to the initial frame since not the displacement and rotations of the structure but the fluctuation of the atoms are considered here.

Obtaining PCs for MD trajectories includes two important stages:

i) generation of the covariance matrix (C),


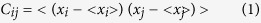


ii) the diagonalization of the 3N × 3N covariance matrix C that can be calculated by eigenvalue decomposition (EVD)^40^,^41^ as


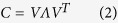


where *Λ* describes the diagonal matrix containing the eigenvalues and *V* describes a matrix including eigenvectors. The eigenvalues display the mean squared displacements (MSD) of atoms (C_α_ atoms in this study) throughout the used eigenvector. In this study, PCA was applied utilizing the bio3D package^42^ in R.

### Graphical Facilities

MD trajectory visualizations, plots, profiles and documents for this work were obtained utilizing Visual Molecular Dynamics (VMD) software^33^, PyMOL molecular visualization system program^43^, R statistical program^44^, QtiPlot data analysis and scientific visualization software, LibreOffice powerful office suite, GNU Image Manipulation Program (GIMP), Maestro 9.7 molecular modeling environment^45^ and Mendeley as a reference manager.

## Results and Discussions

In this study, firstly, the effect of rubredoxin incorporation into the position of IC3 on overall protein behavior was investigated by giving special emphasis to the conformational transitions of the amino acid residues in this domain. The representative structure with the lowest RMSD value taken from 0.3 μs MD simulations was superimposed to the X-ray crystal structure and the mismatching domains were analyzed. In order to improve potency of entry blockers as anti HIV drugs, here we have also examined structural and dynamical changes of maraviroc at the CCR5 binding pocket by long MD simulations. The inhibition mechanism of CCR5 and the effect of inhibitors on the protein conformation upon ligand-binding were studied using MD simulations and principal component analyses (PCA).

### MD Equilibration

**Figure 3:**
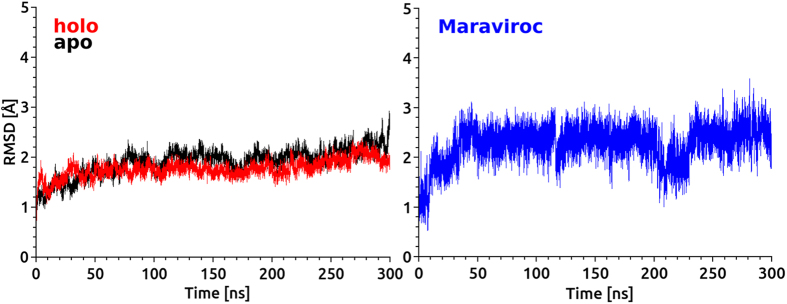
**(left)** The Root mean squared deviations (RMSD) of Cα atoms of comparative apo and holo forms in the course of 0.3 μs MD simulations). **(right)** The RMSD of heavy atoms of the maraviroc during MD simulations.

Root mean square deviation (RMSD) calculations of protein based on backbone atoms as well as RMSDs of heavy atoms of the ligand were performed to determine the structural stability using the RMSD plugin of VMD. It can be seen that both apo and holo forms have reached stable conformations during 0.3 μs MD simulations (Fig. 3). The average deviations of RMSD value for Cα atoms in holo and apo forms were 1.77 Å and 1.97 Å, respectively. The holo form showed slightly higher structural stability compared to apo form. This result is anticipated due to the binding of the inhibitor. RMSD values of the ligand showed that heavy atoms reached to the equilibrium after 30 ns of simulation time and it remained stable with one exception observed in the period of 210 and 230 ns. The first 30 ns trajectory frames were not included in the protein-ligand interaction analyses.

### Fusion Facilitates Influence on CCR5 Conformer

**Figure 4:**
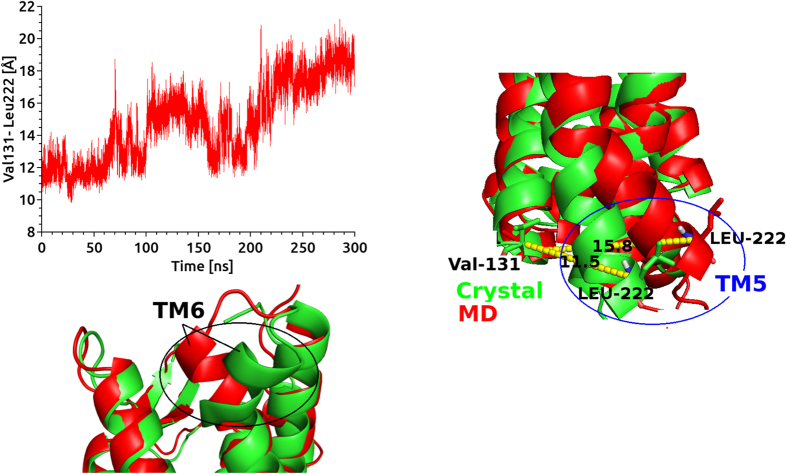
**(left)** Distance analysis between Val131 (cytoplasmic side of TM3) and Leu222 (cytoplasmic side of TM5). **(right)** Superimposition of representative structure from MD (red) with crystal structure (green) near cytoplasmic side of TM5 and TM3. Distances between Val131 and Leu222 are 11.5 Å and 15.8 Å for crystal and representative structure, respectively. **(bottom)** Superimposition of representative structure from MD (red) with crystal structure (green) near EC side of TM6.

Obtaining of well-diffracting X-ray structures of GPCR is one of the most challenging issues in GPCR studies^46^. So far limited number of GPCRs were identified using crystallization methods^46^. A strategy by Kobilika *et al.* was suggested to solve this problem during crystallization of the beta 2 adrenergic receptor (b2AR)^8^. T4 lysozme (T4L) was fused to the cytoplasmic side which was connected TM5 and TM6 domains. Fused T4L is effective to other seven TM helical domains^47^,^48^,^49^,^50^,^51^,^52^,^53^. Rubredoxin, cytochrome b562 RIL (Bril, bRlL, BRIL) and flavodoxin molecules are also used as inserted molecules^54^. In this study, the crystal structure involving rubredoxin as fused partner in cytoplasmic side between TM5 and TM6 was used. Firstly, in order to understand the effect of rubredoxin on CCR5 conformer, we removed the rubredoxin from the crystal structure and missing amino acid residues of IC3 namely, Cys224, Arg225, Asn226 and Glu227 were engineered by homology modeling studies. Holo and apo states of the CCR5 without rubredoxin were then subjected to long MD simulations of 0.6 μs in total. We mostly focused on conformational changes on TM5 and TM6 domains which were more associated with IC3 domain. Besides, two residues, Val131 (TM3) and Leu222 (TM5) present in the terminal domains of cytoplasmic side were also considered. The distance between these residues were highlighted and their evolution distances from each other was plotted (Fig. 4). During the first 100 ns of the simulations, no significant change in the distance between two the residues was observed. In the second 100 ns simulations, TM5 (cytoplasmic end) started to change its conformation and the distance between two terminal residues changed by 4 Å. During third 100 ns simulations, the distance between two terminal residues reached to 8 Å (Fig. 4). A representative structure of 3 × 10^4^ MD trajectories was generated and superimposed with crystal structure of CCR5 containing rubredoxin molecule (Fig. 4). Change in the positions of terminal residues throughout the simulations was shown in Fig. 4. Superimposition of crystal structure with representative structure from MD highlighted another distinct displacement in the EC domain of TM6 (Fig. 4). We showed that the rubredoxin incorporated in the crystallization stage affected the conformation of target structure CCR5. Thus, usage of the TM domain of CCR5 without any refinement via MD simulations might lead misleading results. We believe that the derived target structure after long MD simulations carried out in this work, provide a reasonable target for further investigations of drug-receptor interactions (available upon request from durdagilab.com).

In the rest of study, interactions between CCR5 and maraviroc as well as conformational changes of the target upon inhibitor binding were investigated. In order to elucidate the effect of the drug, a control system (using same MD protocols with holo system) was also prepared for the apo state and MD simulations were carried out.

### Non-bonded Interactions

**Figure 5:**
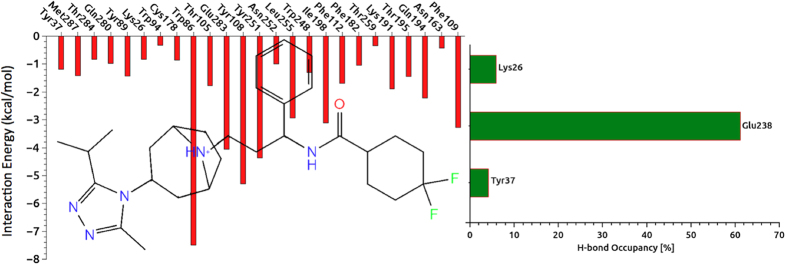
**(left)** Non-covalent bond interactions between the amino acid residues at the binding pocket with the inhibitor. In order to identify individual amino acid residues participate in interaction with each segment of the ligand, the 2D chemical structure of maroviroc was inserted into the profile. **(right)** The occupancy in percentage of each residue participating in H-bonding with the inhibitor.

Non-covalent bond interactions including H-bond interactions between amino acid residues and the inhibitor at the binding pocket were estimated (Fig. 5). In addition, hydrogen bond (H-bond) occupancy value as percentage was identified by applying the cut-off criteria (i.e., distance ≤3.0 Å; angle ≥120 degree) for the proton donor and acceptor atoms. As displayed in Fig. 5, Glu283 at the binding pocket makes a strong and populated (61.1%) H-bond throughout the 0.3 μs MD simulations. The per-residue interaction analysis performed for the CCR5-maraviroc complex system using 3 × 10^4^ trajectories (Fig. 5) showed a good agreement with previously applied experimental studies in which the importance of Glu283 in the stabilization of the complex has been also pointed out^55^,^56^,^57^,^58^,^59^.

**Figure 6:**
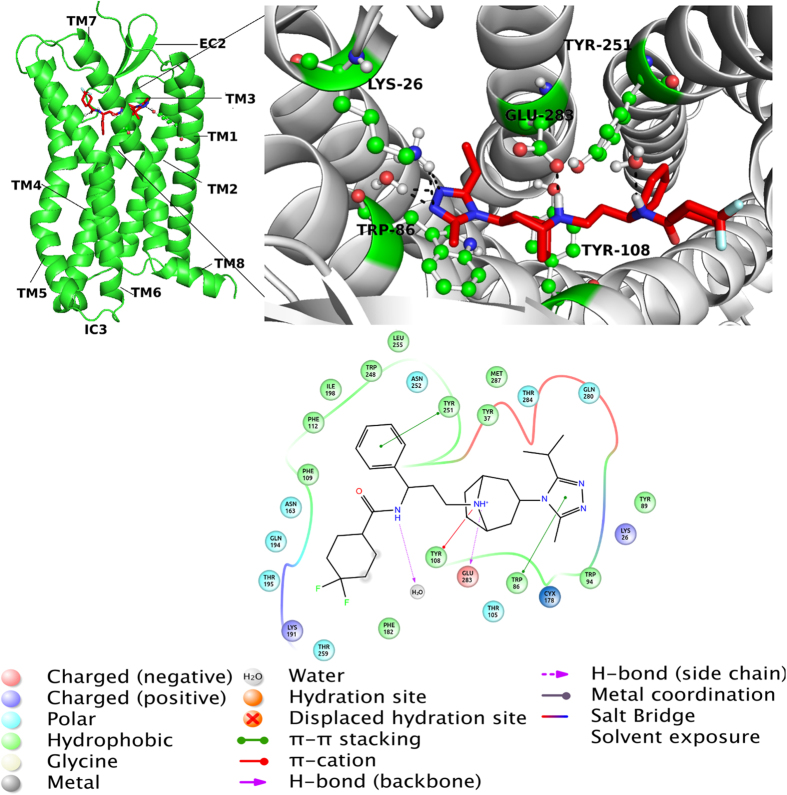
**(left)** Representative complex from 30000 derived frames after MD simulations has been shown. **(right**) 3D ligand interaction diagram show key interactions at the binding site. **(bottom)** 2D ligand interaction diagram represents types of interactions (amino acid residues within 4 Å are considered). Details of interactions are included.

We observed that neither the residues Lys26 and Tyr37 nor Tyr89 and Tyr251 at the binding pocket could not form stable H-bonds with the inhibitor as indicated with low occupancy degrees of Lys26 and Tyr37 as 4.3% and 8.8%, respectively. Figure 6 shows the representative structure of CCR5-maraviroc complex system averaged from the 3 × 10^4^ MD trajectories. While Tyr251 constructed a π-π stacking type interaction with the inhibitor, the nitrogen atom of the tropane group of maraviroc formed a stable H-bond with the Glu283 at the binding pocket. These two interactions between CCR5 and maraviroc were suggested as key interactions for the anti-HIV treatment of the antagonists by Imamura *et al.*^60^ The binding cavity of the CCR5 is mainly hydrophobic because of existence of several nonpolar amino acids (i.e., Trp86, Trp94, Phe109, Phe112, Phe182, Thr195, Ile198, Trp248, Leu255 and Met287)^61^,^62^,^63^. The binding of gp120 to CCR5 depends on several nonpolar amino acid residues such as Trp86, Trp94, and Trp248^61^,^62^,^63^. In the protein-ligand complex, the ligand placed at the bottom part of CCR5 binding cavity which was identified by key amino acids from membrane helices TM1 (Ile28-Asn57), TM2 (Met64-Gln93), TM3 (Asn98-Val130), TM5 (Lys191-Gly216), TM6 (Arg230-Leu255) and TM7 (Gln277-Tyr297). In addition to EC loops of CCR5, amino acid residues in the TM domains (i.e., Tyr37) are included in the linking of gp120^64^. Tyr37 plays an important role in all CC-chemokine proteins and our results (Fig. 6) showed the presence of Tyr37 at the binding pocket which is consistent with the experimental results.

**Figure 7:**
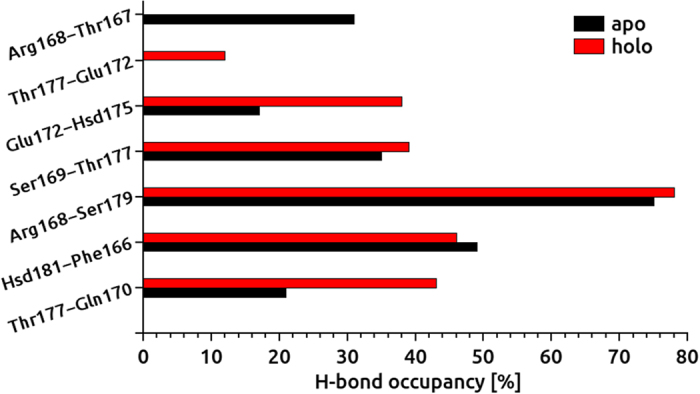
H-bond occupancy of second extracellular loops (EC2) amino acids residues in percentage. A comparative analysis is performed for two states.

Although the inhibitory mechanism of CCR5 is not fully understood so far, an experimental study reports that inhibitors block the HIV entry via allosteric regulation^65^. CCR5 inhibitors are placed in the binding cavity formed by the TM domains close to the EC side of CCR5. The gp120 links to the outer surface (EC loops) of CCR5^66^. EC loops of CCR5 plays a critical role in HIV entry^66^. In order to see the effect of HIV inhibitor on the conformation of EC2 loops, H-bonding occupancies between amino acid residues at this loop were investigated during the simulations (Fig. 7). Although an H-bond was established between Thr167 and Arg168 in the apo state, this bond was not observed at the holo state. On the other hand, although another H-bond was formed between Thr177 and Glu172 at the holo state, this bond did not exist at the apo form.

H bonds between Glu172 and His175 as well as Thr177 and Gln170 were more stable at the holo state. In the absence of maraviroc, %H-bond occupancies between these amino acids decreased dramatically. H-bond occupancies for other amino acid residues at the EC2 were similar for holo and apo states. The average H-bond occupancy at the EC2 loops were observed as 36.5% and 32.5% for holo and apo states, respectively.

Different studies in literature have suggested that the second (IC2) and third intracellular loops (IC3) of GPCRs are the main domains for constructing interactions with G proteins. The amino acids in these domains are mainly responsible for G protein linkages^67^. Recently, an additional proof was reported that the first IC loop is not mainly included in the linking of chemokine receptors to G proteins^67^. GPCR activation process identified by the flexibility and movement of its TM near the cytoplasmic side and three intracellular domains^67^.

Representative structures (i.e., the frame that has the smallest RMSD from average structure) of apo and holo states from each 3 × 10^4^ MD trajectory frames were superimposed. (Supplementary Materials, Fig. S1) A distinct difference between apo and holo states were observed at the EC2 domain. It was shown the influence of maraviroc on EC2 conformer which was critical for gp120 binding. Effect of inhibitor on amino acids at the binding pocket was also examined and illustrated (upplementary Materials, Fig. S1). Not only side-chains, but also backbone topologies of amino acid residues were affected from the inhibitor binding. Particularly Lys26 and Phe182 in holo state have been departed from apo partner. In the position of TM5-IC3 linker (cytoplasmic side of TM5), apo conformer deviated significantly compared to holo conformer (Supplementary Materials, Fig. S1). In order to show the conformational change, we focused on the distance between TM5 and TM6 in the cytoplasmic side for both apo and holo forms. Two amino acids (Val131 from TM3 and Leu222 from TM5) were selected and the distance between them was plotted during the simulations. (Supplementary Materials, Fig. S2) In the first 60 ns, the distance of 12 Å in the holo form did not change whereas in the apo conformer, it started to change after 40 ns and increased about 8 Å at the end of 0.3 μs of simulation time. Although both states showed similar distances between two residues at the end of the simulations, their distance paths during the simulation were not the same (Supplementary Materials, Fig. S2).

### Root Mean Square Fluctuation (RMSF) Calculations

**Figure 8:**
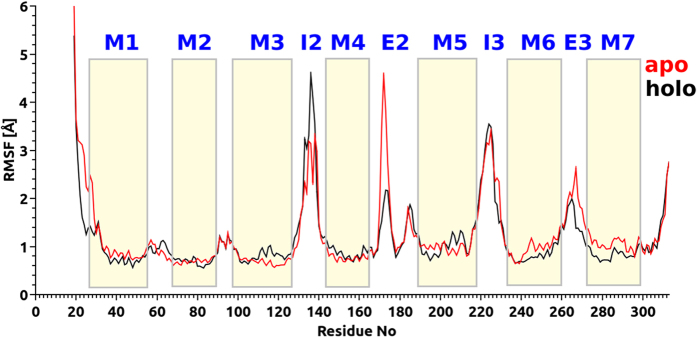
RMSF values of Cα atoms versus residue numbers during 0.3 μs MD simulations. Fluctuation of each TM and loop domains are indicated and compared between the ligand bound- and ligand-free CCR5 chemokine.

In order to investigate the effect of inhibitor on the overall CCR5 target, average fluctuation values were followed for individual amino acid residues during the simulations. 3 × 10^4^ trajectory frames were used in the RMSF calculations. While TM helices showed high stability for both apo and holo states (around 1.0 Å RMSF), IC and IC linkers showed higher fluctuation values (up to 4.0 Å) (Fig. 8). It is well known that N terminus domain at the co-receptor (i.e., Thr171, Glu172 and Lys173 amino acid residues at the EC2 domain) is crucial at the gp120 binding and this region undergoes conformational change before HIV entry to the nucleus. EC2 region of the target structure fluctuated more in apo state (~4.0 Å) compared to holo state (<2 Å). TM1 and TM2 were stable in both states and the inhibitor did not affect the topology of them. However, higher fluctuations were observed in cytoplasmic regions of TM3 and IC2 linkers in the presence of maraviroc. RMSD plots for EC2 loop based on Cα atoms for apo and holo states are given in Fig. S3 of the Supplementary Materials.

### Clustering Analysis

**Figure 9:**
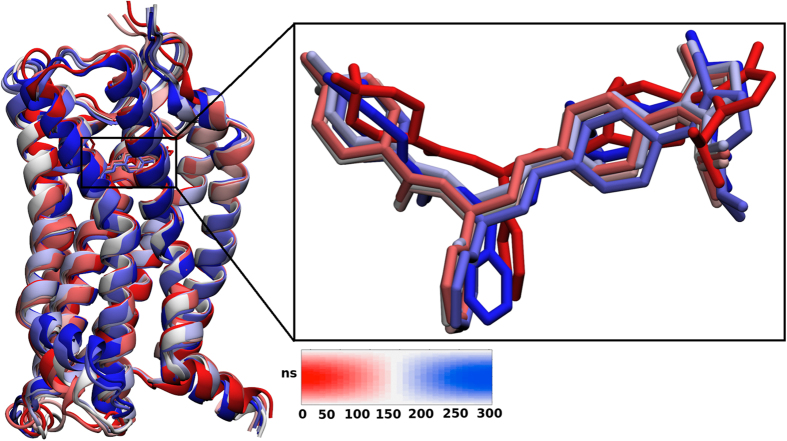
Clustering analysis of the maraviroc conformation into the CCR5 chemokine cavity during MD simulations. A color code is inserted (red: initial, blue: final frames).

Clustering analyses for maraviroc antagonist at the binding cavity of CCR5 was performed to characterize the energetically favored conformations of the drug within the 3 × 10^4^ trajectories. The criterion applied for these analyses was 2 Å RMSD for the heavy atoms of the ligand. A relatively high stability of the ligand was observed during the simulations (Fig. 9). RMSD plots for apo and holo states are given in Fig. S4 of the Supplementary Materials. The higher RMSD values for the amino acid residues of TM5 and EC2 in the holo state were observed.

### Dynamic Cross-Correlations

A cross-correlation analysis was utilized to understand the correlations of the atomic motions in different domains. Dynamic cross-correlation map (DCCM) analysis based on of Cα atoms in apo and holo states of CCR5 was carried out (Fig. S5, Supplementary Materials). The following correlations were observed: i) correlated motions between amino acid residues in the EC domains of TM1, TM2 and TM3 positions and residues in TM5 in the holo state which were not observed in the apo state; ii) strong anti-correlated motion between the residues at TM2, TM3, TM4, EC2 and residues at TM7 and TM6 were observed only in the apo state and attributed to the flexibility of the residues in this state.

### Principal Component Analysis (PCA)

**Figure 10:**
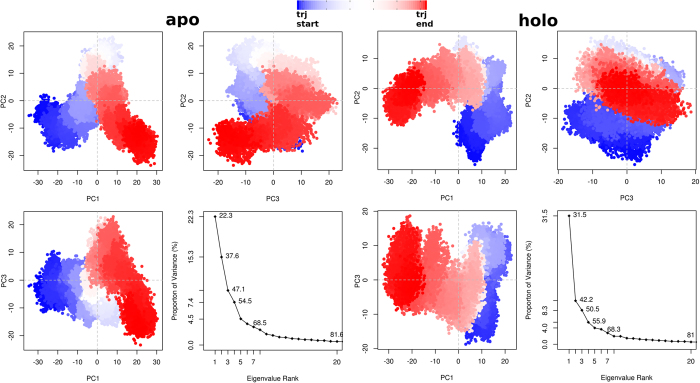
Clustering profiles of average-linkage algorithm on different subspace dimensions projected by first three PCs. Clustering was performed on the overall (except first 20 ns) MD trajectory frames. Percentage of increasing eigenvector participation to the overall CCR5 motion by the apo and holo state. Color scale tracks the trajectory numbers from blue to red.

In order to describe the total norm of receptor motion, we performed PCA based on Cα atoms. This approach assisted to determine the overall combined motion of the Cα atoms in the protein denoted by the eigenvectors of the covariance matrix which is argued by its coincident eigenvalues^68^. The frequency of the eigenvectors with large eigenvalues can usually represent the total concerted motion of the protein correlated to the protein function. The increasing sum of eigenvalues as a function of number of eigenvalues derived from the collected MD frames during 0.3 μs has been shown in Fig. 10. The eigenvalue spectrum showed the proportion of variances versus the eigenvalues for apo and holo systems. Scores for the first PCs for apo and holo forms were 22.3% and 31.5%, respectively. Scores for the second PCs for apo and holo states were 15.3% and 10.7%, respectively. The third PCs for apo and holo systems were 12.8% and 8.3%, respectively. The first three PCs cover 50.4% and 50.5% of the overall motion for apo and holo systems, respectively.

### Conformational Clustering Respect to Main PCs

**Figure 11:**
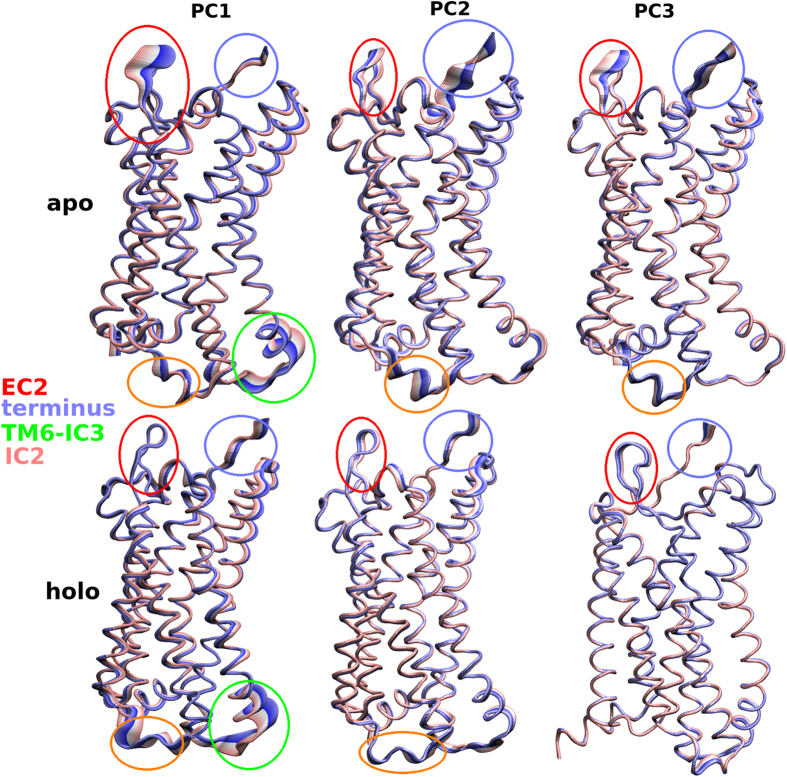
Domains, directions and degrees of motions respected to the first three PCs were illustrated. The main motion are indicated by broadened tubes (color was changed with the course of time step). First and second columns display the apo and holo state of CCR5, respectively.

Although PCA displayed the important motions of the proteins at the apo and holo states, it did not split the frames into different structural groups. Various of the protein structures are clustered in subsets according to their structural similarity^69^. In the present work, 3 × 10^4^ conformers for both apo and holo states were clustered using the first three PCs (Fig. 10). Clustering of generated conformers were plotted in three cross profiles, PC1-PC2, PC1-PC3 and PC2-PC3 for both systems. The side-chains were excluded in this analysis and only Cα atoms were considered. The distribution of dots (frames) throughout the *x* and *y* axes, represented the comparative contribution to the whole dynamic from each PC. Although regular and organized set of conformations were observed in the cross-plots, the conformational discrepancies of apo and holo forms due to the presence of the inhibitor molecule, were clearly seen. In Fig. 11, atomic deviations in the first three PCs during the simulations were visualized. In rows 1, 2 and 3, conformers of PCs were overlaid for both systems. Broadened tubes in these profiles displayed displacement, while slim tubes displayed domains that stayed almost rigid. Dynamical variations between apo and holo states can be clearly shown from the motion of Cα atoms. The flexibility in EC2 domain in apo protein was also seen here and this results is consistent with the results obtained from the PCA. It is clear that the presence of the inhibitor had a main influence on the conformational space of EC2 in complex.

## Conclusions

In this study, post-processing MD analysis including PCA and clustering approach for a marketed drug was performed on derived trajectories via long MD simulations. To the best of our knowledge, this work presented here is the most exhaustive study on CCR5-inhibitor interaction based on the high-resolution crystal structure. We showed that the rubredoxin co-crystallized with the protein had a significant impact on the structure especially on the cytoplasmic side of TM5. The gp120-co-receptor activity was inhibited by anti-HIV drug maraviroc and its inhibition role on EC2 was identified with long MD simulations. The importance of EC2 against HIV infection was pointed out. The critical residues (Thr171, Glu172 and Lys173) taking part in the inhibition mechanism were identified. Our theoretically predicted results are shared a good agreement with experimental studies as Dogo-Isonagie *et al.* and Abayev *et al.*^70^,^71^ have suggested that EC domains play a key role in HIV fusion. They tried to produce peptides based on EC2 loop of CCR5 and also their biochemical studies have showed the importance of EC domains in binding gp120 and mediating viral entry. Another study performed by Lee *et al.*^72^ also approves our theoretically obtained results. They illustrated the influence of EC2 at inhibition of gp120-CCR5 binding by generating a large panel of anti-CCR5 monoclonal antibodies. Zhang *et al.*^73^ experimentally described the impact of specific amino acid residues (i.e., Thr171, Glu172) in anti-HIV treatments, which our computational results also showed the importance of these residues in inhibition mechanism. The results obtained from the PCA and MD trajectory analyses highlighted the important structural and dynamical differences between the apo and holo forms of the CCR5. We believe that our results will be a guide for the future simulations related to HIV inhibition studies and will lead to discovery of novel potent antagonists inhibiting CCR5.

## Additional Information

**How to cite this article**: Salmas, R. E. *et al.* Investigation of Inhibition Mechanism of Chemokine Receptor CCR5 by Micro-second Molecular Dynamics Simulations. *Sci. Rep.* **5**, 13180; doi: 10.1038/srep13180 (2015).

## Supplementary Material

Supplementary Information
