# Supplementary material for: Investigation of Inhibition Mechanism of Chemokine Receptor CCR5 by Micro-second Molecular Dynamics Simulations
Source: Sci Rep. 2015 Aug 24;5:13180. doi: 10.1038/srep13180 (PMC4547396; doi:10.1038/srep13180)
Supplement: Supplementary Information [file srep13180-s1.doc]

# Supporting Information

**Investigation of Inhibition Mechanism of Chemokine Receptor CCR5 by Micro-second Molecular Dynamics Simulations**

Ramin Ekhteiari Salmas^†^, Mine Yurtsever^†^, Serdar Durdagi^‡^

^†^Department of Chemistry, Istanbul Technical University, Istanbul, Turkey

^‡^Department of Biophysics, School of Medicine, Bahcesehir University, Istanbul, Turkey


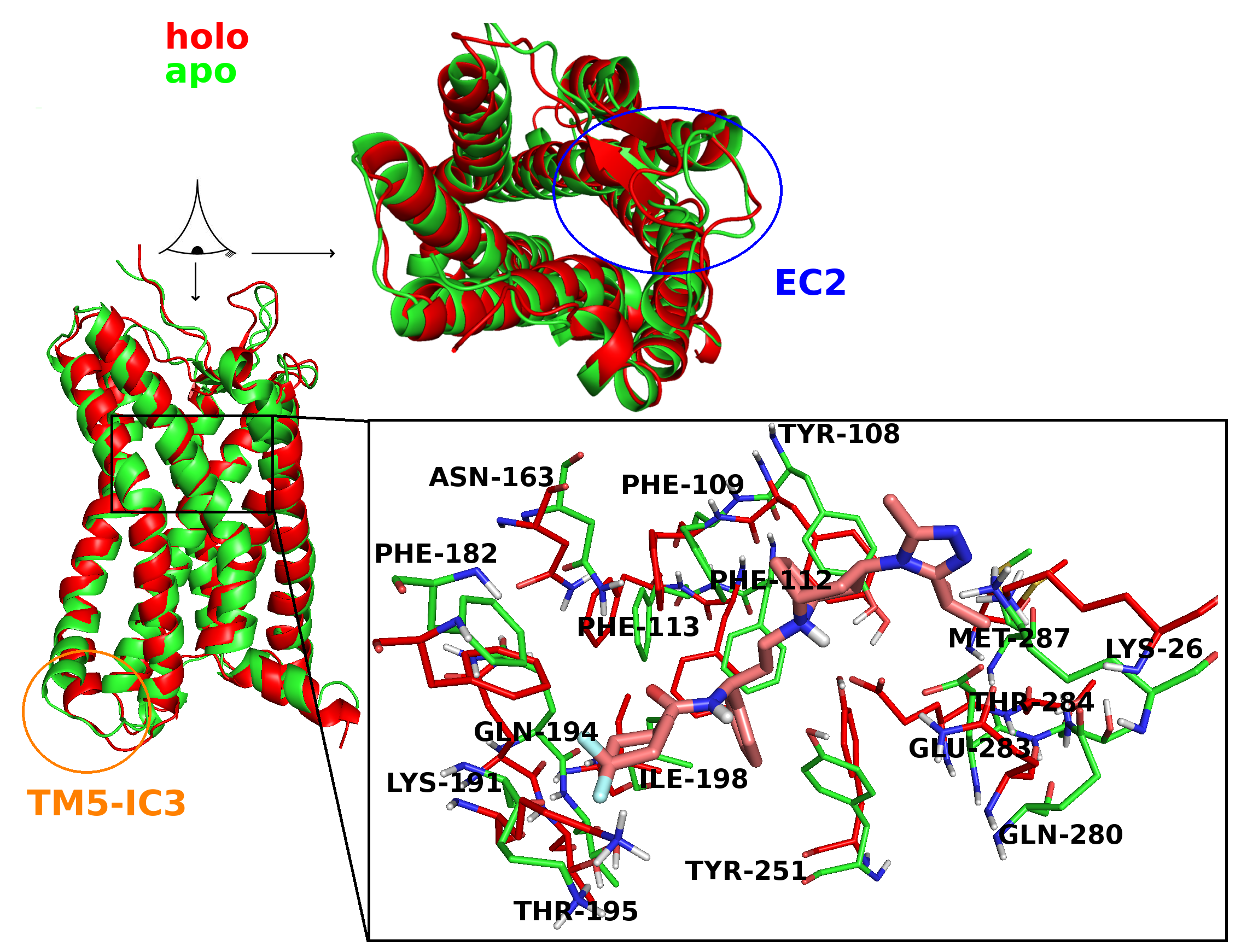


**Figure S1.** Superimposition of representative apo and holo forms. Upper panel shows extra-cellular side and lower panel display 7TM active cavity.


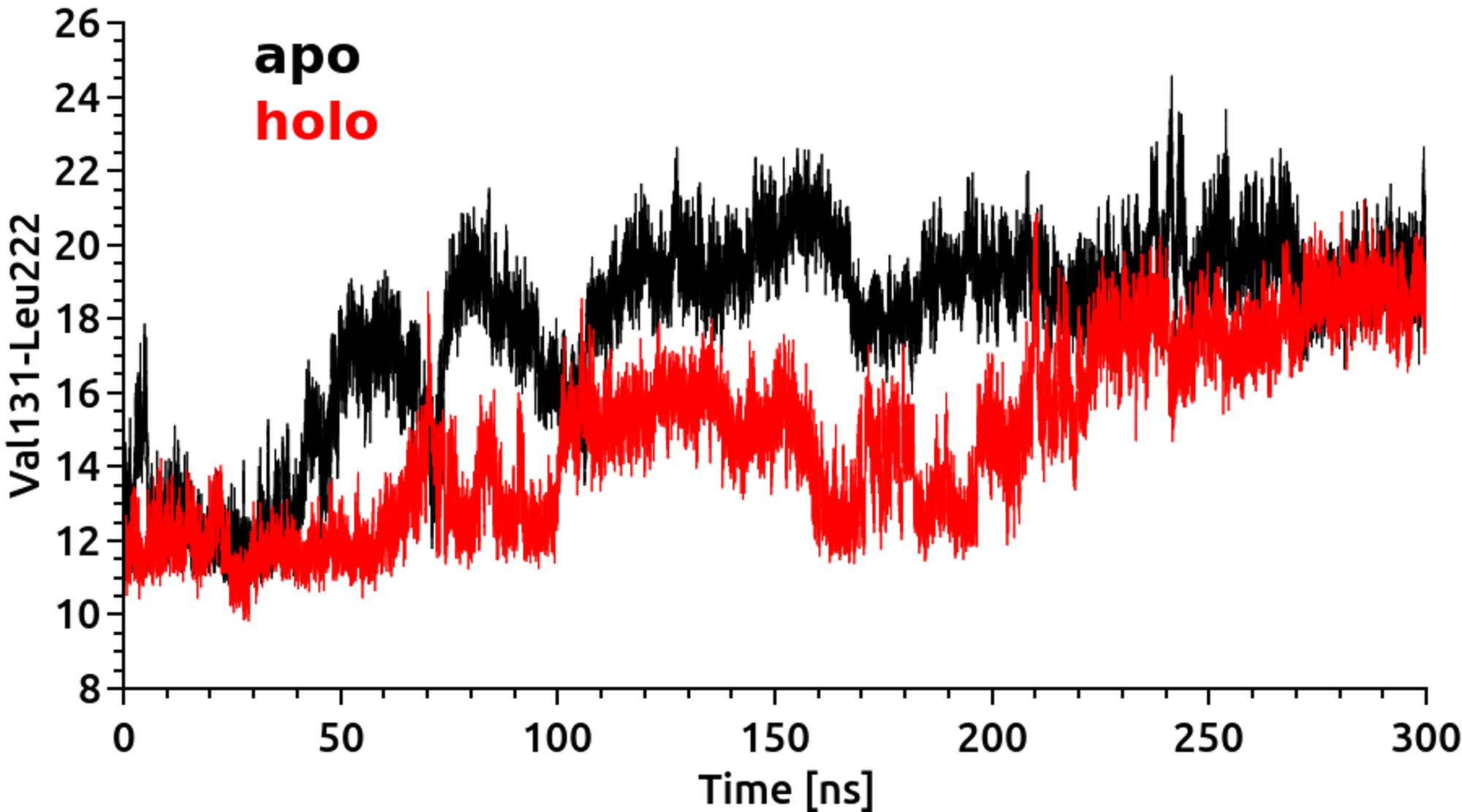


**Figure S2.** Distance plot shows the distance evolution between Val131 (TM5) and Leu222(TM6) amino acid residues respect to MD time.


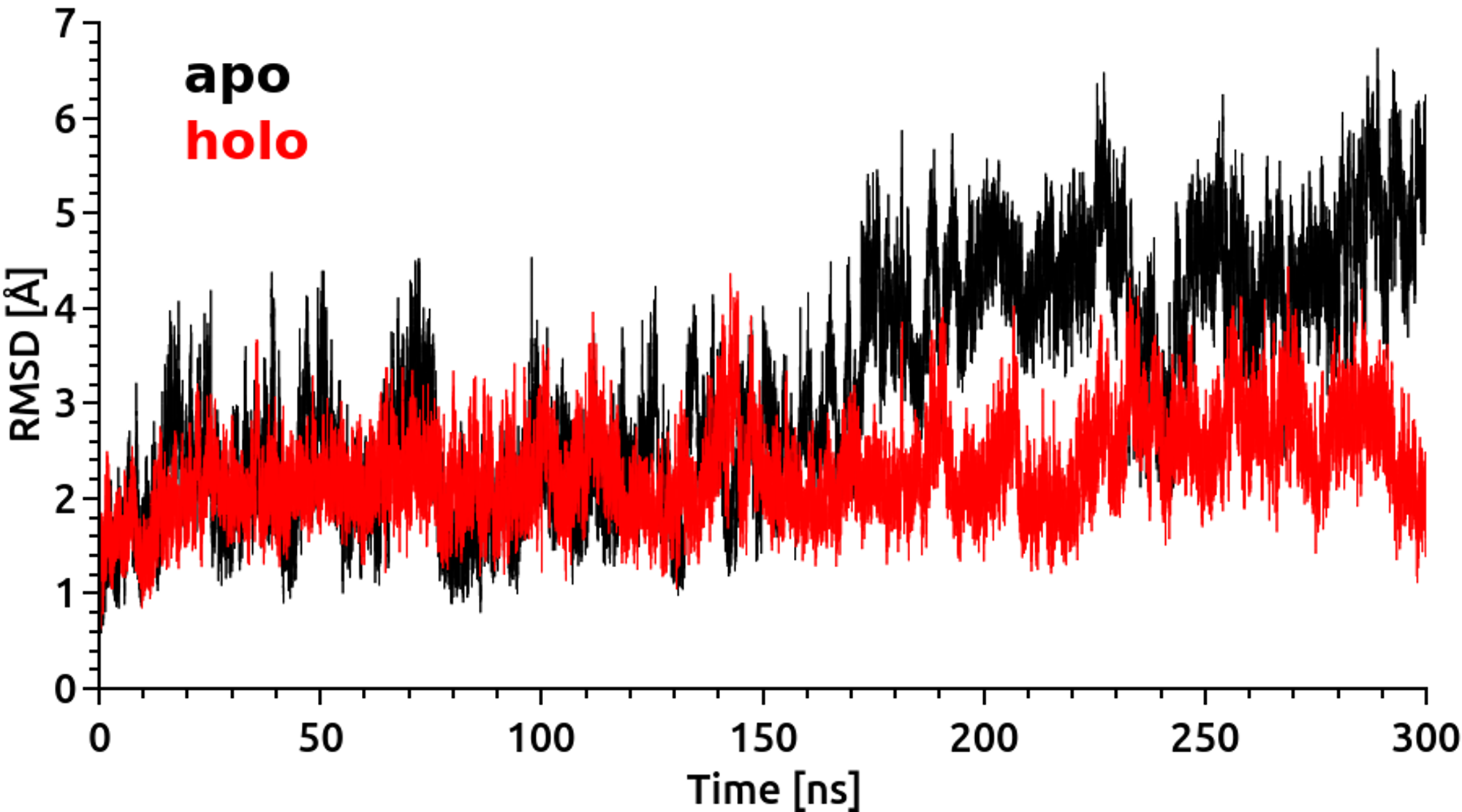


**Figure S3.** RMSD evolution of EC2 amino acid residues with the base of Cα atoms for apo and holo systems.


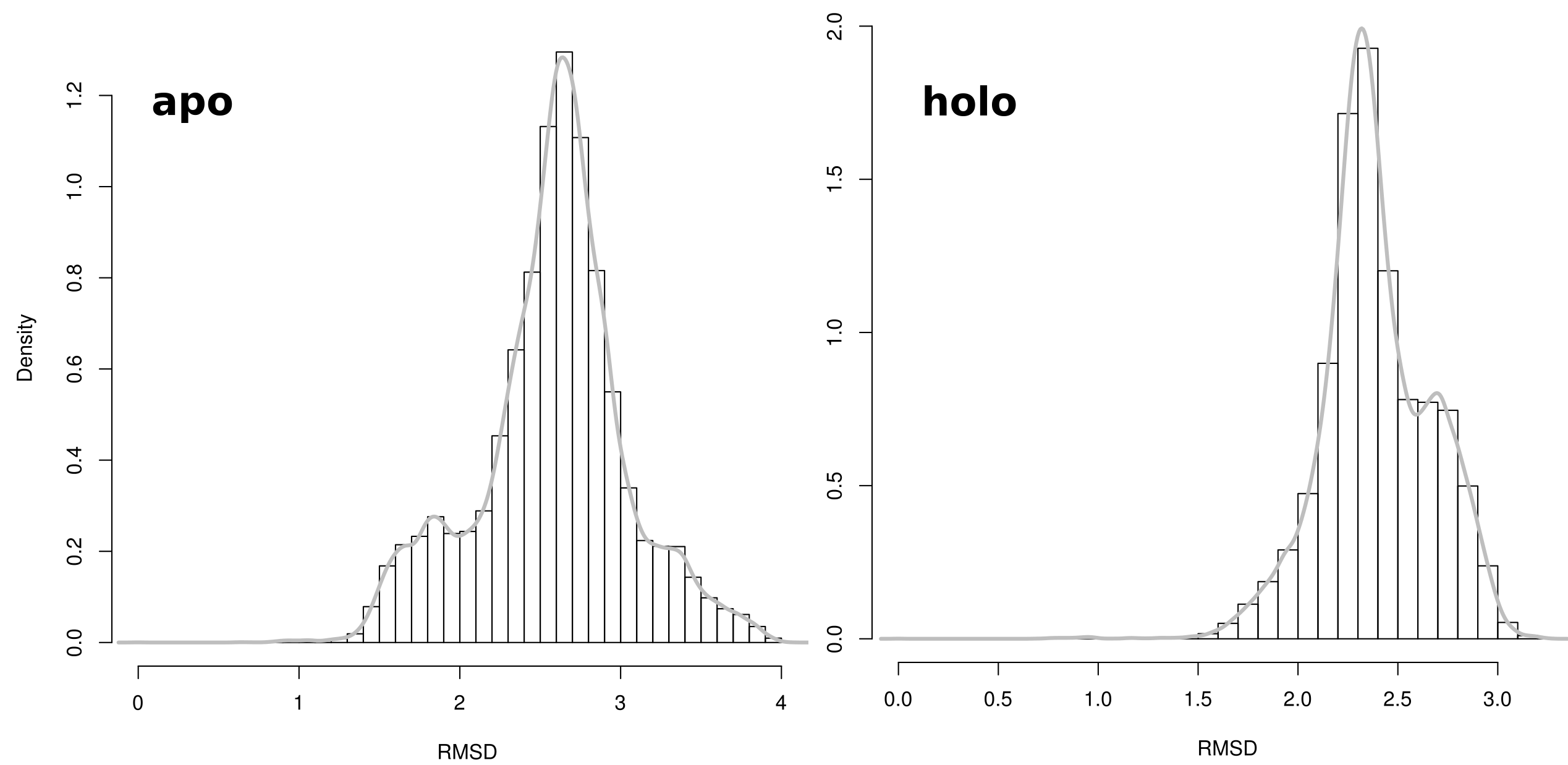


**Figure S4.** Histograms over Cα atoms RMSD values (Å).


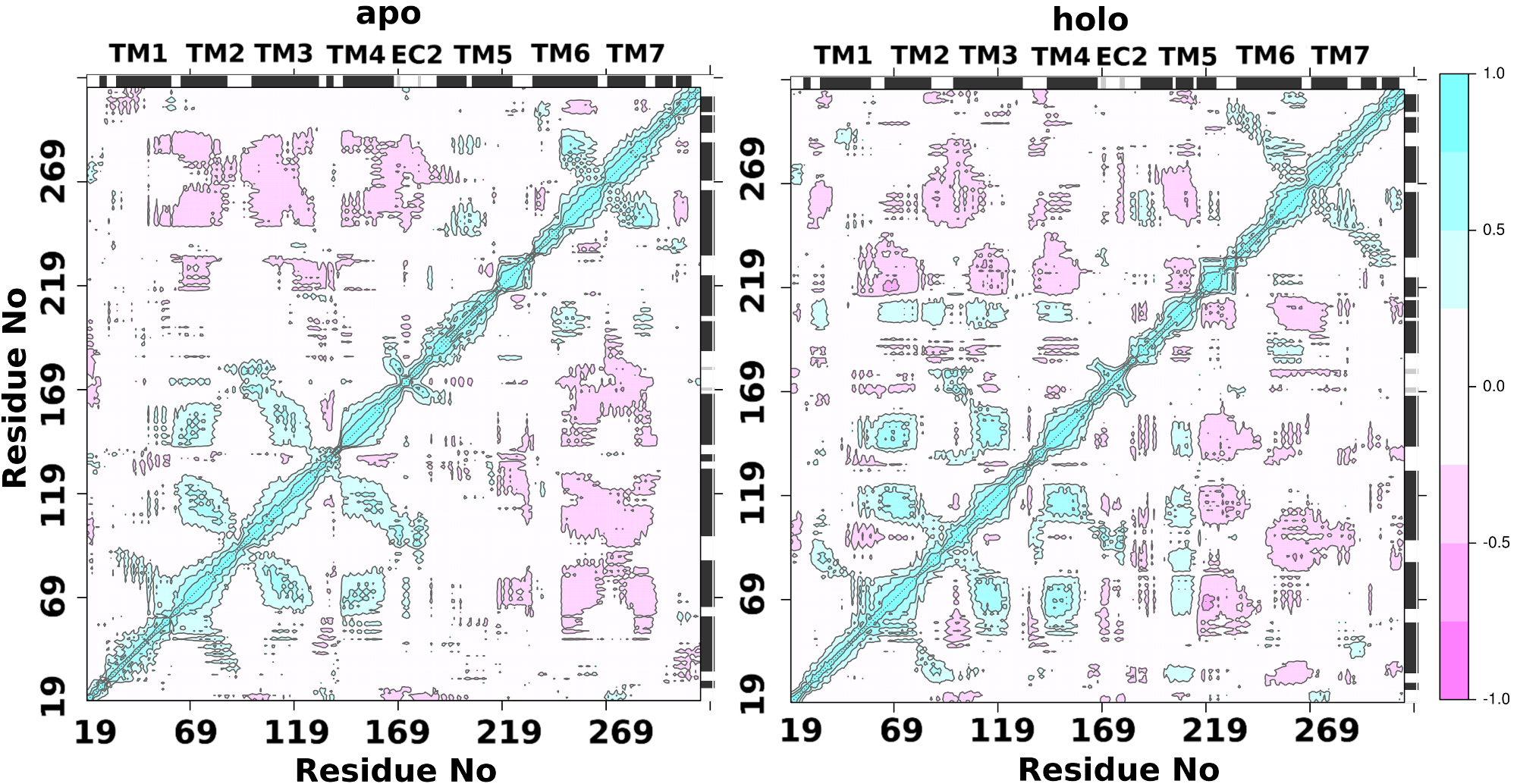


**Figure S5.** Cα-residue dynamics cross correlation map for the ligand-free and ligand-bound CCR5. The color measure operates pink (-1 to -0.75) to white (-0.25 to 0.25) to cyan (0.75-1). Negative amounts (pink) show that Ca atoms displace in opposite way, called anti correlated motions, but positive amounts (cyan) display correlated motions happening at the one way.
